# Supplementary material for: Family care and predictors of the disabled elderly in China: A cross-sectional study based on the Anderson model
Source: PLoS One. 2024 Nov 4;19(11):e0312002. doi: 10.1371/journal.pone.0312002 (PMC11534204; doi:10.1371/journal.pone.0312002)
Supplement: Attachment — Submitted filename: Response to Reviewers.docx [file pone.0312002.s001.docx]

**Response Letter**

Dear editors and reviewers,

We are very grateful for your constructive comments and suggestions for our manuscript entitled" Family care and predictors of the disabled elderly in China: a cross-sectional study based on the Anderson model"(lD:PONE-D-24-18004).Your comments are very valuable and helpful for improving our manuscript. In the following the responses to all the comments are provided one by one.We have tried our best to make all there visions clear, and we hope that the revised manuscript can satisfy the requirements for publication.The main revisions in the new manuscript are:

1. More specific expressions have been used;
2. Abstract has been updated;
3. Introduction has been supplemented.

Sincerely,

Corresponding author.

**Response to the comments of Reviewer #1**

Q1. The manuscript was not presented in an easily understandable manner and written in standard English.

Thank you for your insightful question, line 25: aging and aging has been changed to “In light of China's progressively aging population” , line 146: slightly,line149: average, in table 1: moderate,line150: poor: in table 1 mild have been changed to “Mild disability”、“Moderate disability”、“Severe disability”

Q2. The main problem of this study is that the results are very much about who is providing family care (spouses and children). No information is given on the burden that family members might experience in providing care. In the discussion it is mentioned "family care is difficult ..." and that family member are at risk of mental and physical health detoriation. However, there are no results that substantiates these conclusions.

Thank you for your insightful comment and kind suggestion. Family members might experience in providing care has been supplemented. "family care is difficult ..."has been deleted.

Q3. The abstract is not always clear. The abstract is not always clear line 25: aging and aging .. what does this mean?line 38-40: the conclusion is not supported by the data.

Thank you very much for the constructive feedback.The conclusions have been modified in the light of the results of the study.

Q4.Introduction: line 18, 19: this gives the impression that elderly disabled have to rely on informal care since formal care cannot meet the demands.line 59: 60+? 65+.line 61: however ?.line 68-70: sustainability ... is under threat. If this is the heart of the research than other data needed to be accumulated, i.e. on the burden of informal care

Thank you for your remainder.The expression of ambiguity has been clarified, more data about China's elderly population has been included, and a body of literature has been gathered regarding the variables affecting the caregiving burden on families.

Q5. Discussion:176, 177: Family care is difficult: there are no data presented here to support this.180: put spouses and children at risk: no data presented in this research to support this claim.198, 199: specific assistance is required: no data to support this.

We thank the reviewer for the highly valuable comment. Discussions that lacked evidence have been removed.

**Response to the comments of Additional Editor**

Q1. Main issues are the selection of "predictors" for family care, with very much literature available (especially burden of care, further discrimination to different care tasks?) and the embedding of your discussion in both your own results (which should substantiate your conclusions) and literature.

We thank the reviewer for the highly valuable comment.The relevant available literature has been added and the discussion will be embedded in its own results.

The paper has been revised carefully and thoroughly according to the comments from the reviewers. All the questions from the reviewers have been answered.Thanks to the professional comments again that point out the above problems. The authors hope these explanations would answer your doubts.
